# Differential expression pattern of co-inhibitory molecules on CD4^+^ T cells in uncomplicated versus complicated malaria

**DOI:** 10.1038/s41598-018-22659-1

**Published:** 2018-03-19

**Authors:** Annemieke Abel, Christiane Steeg, Francis Aminkiah, Otchere Addai-Mensah, Marylyn Addo, Nicola Gagliani, Christian Casar, Denis Dekugmen Yar, Ellis Owusu-Dabo, Thomas Jacobs, Maria Sophia Mackroth

**Affiliations:** 1 Protozoa Immunology, Bernhard Nocht Institute for Tropical Medicine, 20359 Hamburg, Germany; 2 Kumasi Centre for Collaborative Research in Tropical Medicine, Kumasi, Ghana; 3 Department of Medical Laboratory Technology, Faculty of Allied Health Sciences, Kwame Nkrumah University of Science and Technology, Kumasi, Ghana; 4 I. Medical Department, Division of Tropical Medicine and Infectious Diseases, University Medical Centre Hamburg Eppendorf, 20246 Hamburg, Germany; 5 German Center for Infection Research (DZIF), partner site Hamburg-Lübeck-Borstel-Riems, Germany

## Abstract

The immune response of malaria patients is a main factor influencing the clinical severity of malaria. A tight regulation of the CD4^+^ T cell response or the induction of tolerance have been proposed to contribute to protection from severe or clinical disease. We therefore compared the CD4^+^ T cell phenotypes of Ghanaian children with complicated malaria, uncomplicated malaria, asymptomatic *Plasmodium falciparum (Pf)* infection or no infection. Using flow cytometric analysis and automated multivariate clustering, we characterized the expression of the co-inhibitory molecules CTLA-4, PD-1, Tim-3, and LAG-3 and other molecules implicated in regulatory function on CD4^+^ T cells. Children with complicated malaria had higher frequencies of CTLA-4^+^ or PD-1^+^ CD4^+^ T cells than children with uncomplicated malaria. Conversely, children with uncomplicated malaria showed a higher proportion of CD4^+^ T cells expressing CD39 and Granzyme B, compared to children with complicated malaria. In contrast, asymptomatically infected children expressed only low levels of co-inhibitory molecules. Thus, different CD4^+^ T cell phenotypes are associated with complicated versus uncomplicated malaria, suggesting a two-sided role of CD4^+^ T cells in malaria pathogenesis and protection. Deciphering the signals that shape the CD4^+^ T cell phenotype in malaria will be important for new treatment and immunization strategies.

## Introduction

Malaria remains one of the leading causes of morbidity and mortality among children in Sub-Saharan Africa^1^. An infection with *Plasmodium falciparum* (*Pf*), the most prevalent malaria parasite in Africa, can lead to a wide spectrum of disease manifestations, ranging from severe, life-threatening malaria through mild, febrile illness (uncomplicated malaria) to asymptomatic infections. The immune response of the infected host is one important factor that influences the course of a *Pf* infection. In individuals with little prior exposure, an infection with *Pf* activates a strong, pro-inflammatory response^2,3^, which induces fever and contributes to the development of malaria complications^4,5^. In endemic areas, regularly exposed children gradually develop a partial immunity which protects from severe and febrile disease and is associated with an increasing incidence of asymptomatic infections.

CD4^+^ T cells are an important player of the adaptive immune response to plasmodia and can provide protection but also have detrimental effects and contribute to disease complications^5–9^. Several observations support the idea that qualitative changes of the T cell response occur during acute malaria^10,11^. Murine models with chronic *Plasmodium* infection show that the initial strong pro-inflammatory response is downregulated during the course of the infection^12^. Previous studies in humans and mice by us and others have shown that acute malaria induces an upregulation of co-inhibitory molecules, such as cytotoxic T-lymphocyte-associated antigen-4 (CTLA-4), programmed cell death-1 (PD-1), lymphocyte-activation gene-3 (LAG-3) or T-cell immunoglobulin and mucin domain-3 (Tim-3) on CD4^+^ T cells which leads to impaired cytokine production of the CD4^+^ T cells^13–18^. The blockade of the co-inhibitory receptors CTLA-4, PD-1, Tim-3 and/or LAG-3 leads to an enhancement of the pro-inflammatory T cell responses and a more severe course of disease in murine malaria models, but can also improve parasite clearance, indicating the double-edged role of CD4^+^ T cells in malaria^13,14,16,17^.

Co-inhibitory molecules such as PD-1, LAG-3 or Tim-3 are also preferentially expressed on regulatory T cells including Type 1 regulatory T cells (Tr1 cells), Tr27 and other peripherally induced regulatory T cell subsets, which are expanded during natural exposure or in experimental infection models of malaria^18–22^. Other activation and effector molecules which are expressed on regulatory as well as activated T cells and have been shown to modulate immune responses to infectious pathogens include Granzyme B (GrzB), CD39 or CD38^23–25^.

Most of the CD4^+^ T cell analyses conducted so far have been in murine models or in experimental human malaria infections. It still remains unclear which T cell profiles are associated with clinical protection upon natural exposure in endemic areas. In our study, we therefore compared T cell phenotypes in children with different clinical severities of malaria in an endemic setting and focused on T cell markers with regulatory capacity, using multi-colour flow cytometry analysis and automated multivariate clustering.

Children with complicated versus uncomplicated malaria expressed different CD4^+^ T cell signatures. Children with complicated malaria showed higher frequencies of CTLA-4^+^ and PD-1^+^CD4^+^ T cells, whereas children with uncomplicated malaria had higher percentages of CD39^+^, as well as GrzB^+^CD4^+^ T cells, suggesting that distinct regulatory mechanisms are activated and might shape the clinical picture of acute malaria.

## Results

### Characteristics of study participants

Blood samples were collected from healthy, afebrile children at Jachie Primary School and children with acute malaria at St Michaels Catholic Hospital in Pramso, Bosumtwi District, Ashanti Region in Ghana. 82 healthy, afebrile children between 5–11 years of age were enrolled at the primary school, of whom 41 were not infected with *Pf* (healthy controls = HC) and 41 were asymptomatically infected with *Pf*, detected by HRP2 -rapid diagnostic test (asymptomatically infected = AS). 30% of the screened children at the school were positive for *Pf*. At the hospital, 66 children with acute, symptomatic malaria between 1–12 years of age were enrolled, of whom 32 were treated as outpatients with oral artemisinin combination drug for uncomplicated malaria (outpatient = OP) and 34 were treated as inpatients with iv Artesunate for clinically diagnosed complicated malaria (inpatient = IP). As a consequence of the collection strategy at the school, healthy controls and asymptomatically infected children were on average older than children with acute malaria (Table 1 and Supplementary Fig. S1; a detailed description of the study participants can be found in Supplementary Table S1). All children with acute, symptomatic malaria and 37% (15/41) of the asymptomatically infected children were microscopically positive for *Pf* infection by thin blood smear. The group of children treated as inpatients for complicated malaria showed the highest parasitemia with a mean parasitemia of 4.5% whereas children with uncomplicated malaria had a mean parasitemia of 1.4%. Asymptomatically infected children showed only low parasitemia <1% (n = 15) or no microscopically detectable parasitemia by thin blood smear (n = 26) (Table 1 and Supplementary Table S1).

**Table 1:** Characteristics of study participants.

Group	Number of children	Age [years]	Sex females/total [percent]	HRP rapid test	Thin blood smear	Parasitemia
HC	41	8.5 ± 0.2	27/41(65.85%)	All negative	All negative	—
AS	41	9.1 ± 0.2	24/41(58.54%)	All positive	15 positive/26 not detectable	0.07 ± 0.02 N = 41
OP	32	5.7 ± 0.5	18/32(56.25%)	All positive	All positive	1.43 ± 0.29 N = 24
IP	34	4.7 ± 0.5	16/35(45.71%)	All positive	All positive	4.49 ± 0.79 N = 28

HC = Healthy control; AS = Asymptomatic; OP = Outpatient; IP = Inpatient; HRP = Histidin-rich Protein.

### Higher proportion of CTLA-4^+^ and PD-1^+^ CD4^+^ T cells in children with complicated malaria compared to children with uncomplicated malaria

**Figure 1:**
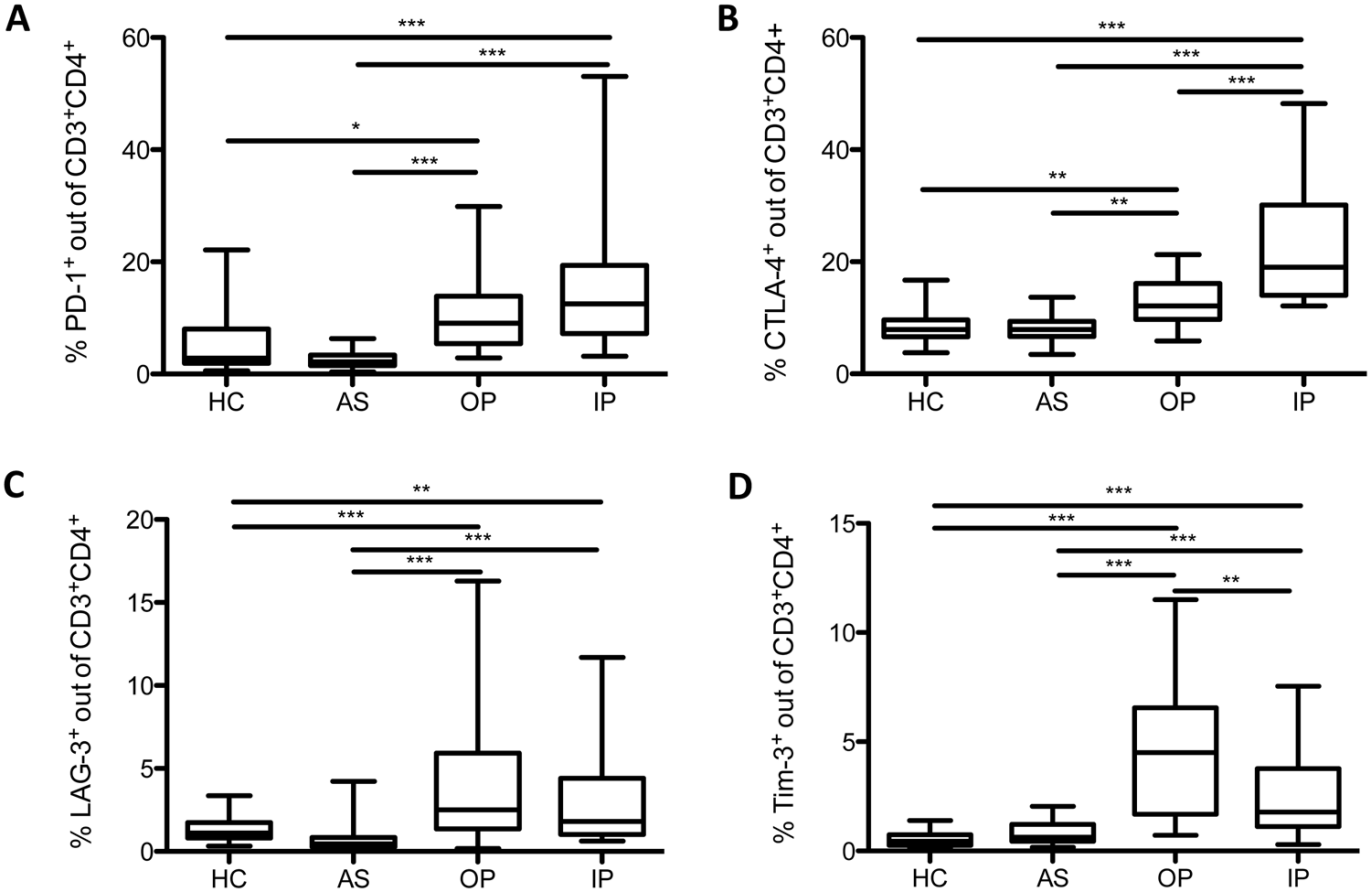
Higher proportions of PD-1^+^ and CTLA-4^+^ CD4^+^ T cells in children with complicated malaria. Blood samples from patients and controls were *ex vivo* analyzed for the expression of PD-1, intracellular CTLA-4, LAG-3, and Tim-3 on CD4^+^ T cells by flow cytometry. Box plots demonstrating expression of PD-1 (**A**), CTLA-4 (**B**), LAG-3 (**C**), and Tim-3 (**D**) in all four study groups. Statistical differences are shown according to One-way ANOVA with Tukey’s Multiple Comparison Test.

We first examined the *ex-vivo* expression of the co-inhibitory molecules PD-1 and CTLA-4 on CD4^+^ T cells in our four study groups. Both groups of children with acute malaria showed higher frequencies of CTLA-4^+^CD4^+^ T cells than uninfected or asymptomatically infected children. The highest percentage was seen in the inpatient group with complicated malaria (Fig. 1B). Similarly, the expression of PD-1 on CD4^+^ T cells was more frequently observed in children with acute malaria and there was a trend towards a higher proportion of PD-1^+^CD4^+^ T cells in children with complicated than uncomplicated malaria (Fig. 1A). Of note, the proportions of CD4^+^ T cells were similar in all four study groups (Supplementary Fig. S2). Next, we assessed the expression of the co-inhibitory molecules Tim-3 and LAG-3 on CD4^+^ T cells. Like CTLA-4 and PD-1, both molecules showed a more frequent expression in children with acute malaria, compared to the school children (Fig. 1C and D). In contrast to the observation with CTLA-4 and PD-1, Tim-3 was more frequently expressed on CD4^+^ T cells in children with uncomplicated versus complicated malaria (Fig. 1D). There was no significant difference in expression levels of LAG-3 between the two patient groups (Fig. 1C). Regarding the expression pattern of co-inhibitory molecules, no significant differences between non-infected and asymptomatically infected children were detected (Fig. 1). The expression pattern of co-inhibitory molecules in children with acute malaria did not differ by age. Similar levels were detected in children <5 years of age and >5 years of age (Supplementary Fig. S3). The proportion of PD-1^+^ and CTLA-4^+^CD4^+^ T cells weakly correlated with parasitemia at time of diagnosis (Supplementary Fig. S4). In contrast, neither LAG-3 nor Tim-3 expression showed a correlation with parasitemia (Supplementary Fig. S4).

### Automated multivariate clustering reveals different T cell cluster in children with complicated malaria compared to children with uncomplicated malaria

**Figure 2:**
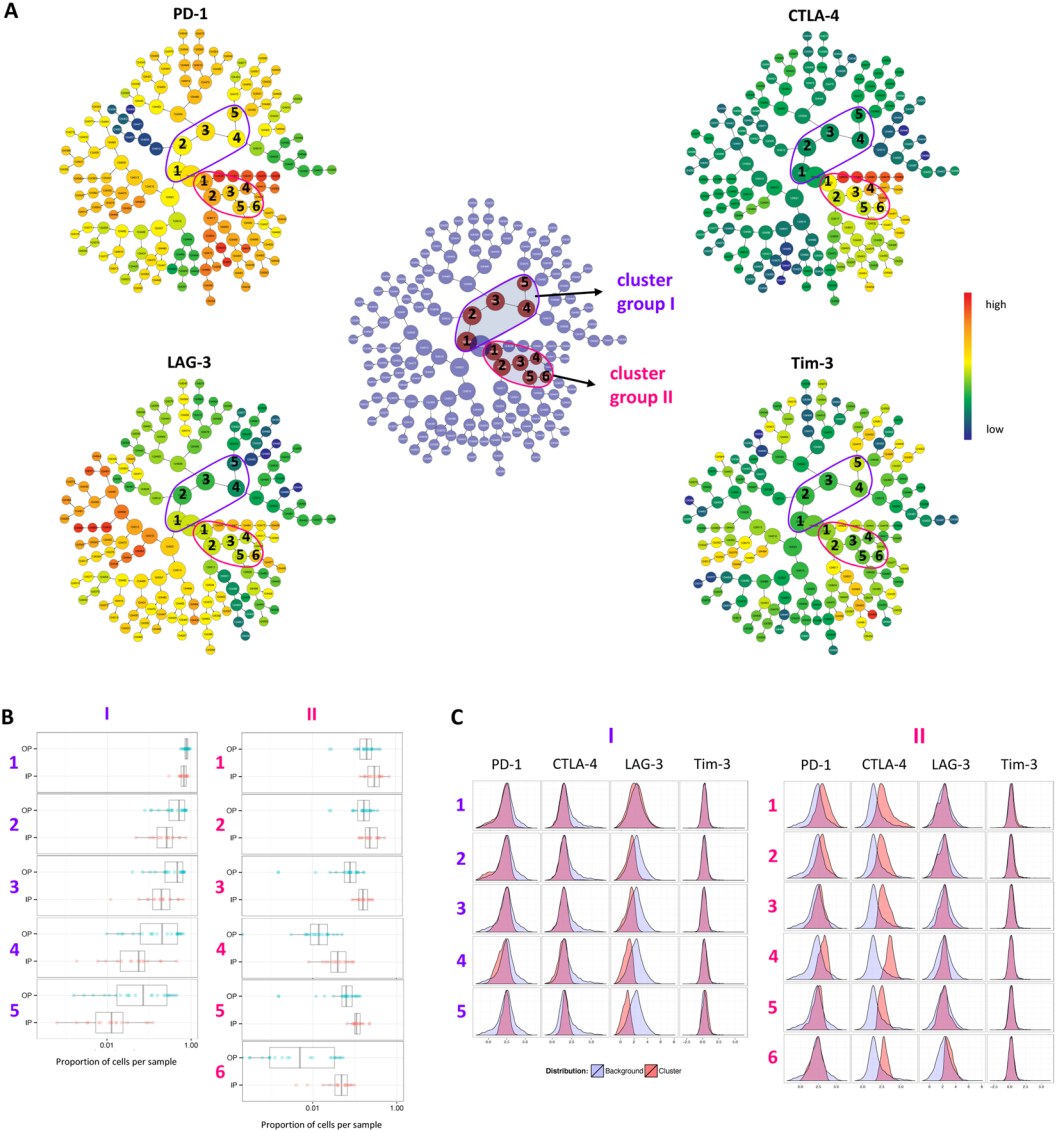
Identification of differing cell signatures between children with uncomplicated and complicated malaria using automated hierarchical clustering. (**A**) CITRUS with group PAMR analysis (R implementation of Prediction Analysis for Microarrays) of PD-1, CTLA-4, LAG-3 and Tim-3 expression of samples from children with complicated (IP) and uncomplicated (OP) malaria. Differing clusters are highlighted in red (centre) and categorized into cluster group I (purple) and cluster group II (pink) (centre). Within each cluster group, clusters are numbered. Four parameter clustering of OP and IP samples demonstrating individual parameter expression intensities of PD-1, CTLA-4, LAG-3 and Tim-3 (outer graphs). Cv.1se was used with a 10 fold cross validation and error rate of 30%. (**B**) Each line represents a numbered cluster defined in A. Abundance of cells within the identified differing clusters in the OP and IP groups. The dots represent samples within the groups. (**C**) Intensity of the expression of co-inhibitory molecules within the differing clusters. Expression of the specific molecules within the total amount of cells is shown in grey, expression within the specific cluster is shown in red.

We next conducted a multivariate analysis of co-inhibitory molecule expression between children with complicated and uncomplicated malaria, using the software CITRUS (cluster identification, characterization, and regression), which allows for an automated identification of stratifying subpopulations. The automated hierarchical clustering of the flow cytometry data of the two groups showed two differing cluster groups of the co-inhibitory molecules analyzed (Fig. 2). Cluster group I showed a lower expression of PD-1 and CTLA-4, compared to background, and was more abundant in children with uncomplicated malaria. Cluster group II showed a higher expression of CTLA-4 and PD-1 and was more frequent in children with complicated malaria than in children with uncomplicated malaria. There were almost no changes in Tim-3 expression, compared to background. LAG-3 was downregulated in cluster group I, indicating a lower expression in children with uncomplicated malaria. In cluster group II, LAG-3 showed a higher than background expression only in cluster 6, signifying a higher expression of LAG-3 for one T cell cluster in children with complicated malaria (Fig. 2A and C).

### Uncomplicated malaria is associated with a stronger upregulation of CD39, CD69, and Granzyme B on CD4^+^ T cells

**Figure 3:**
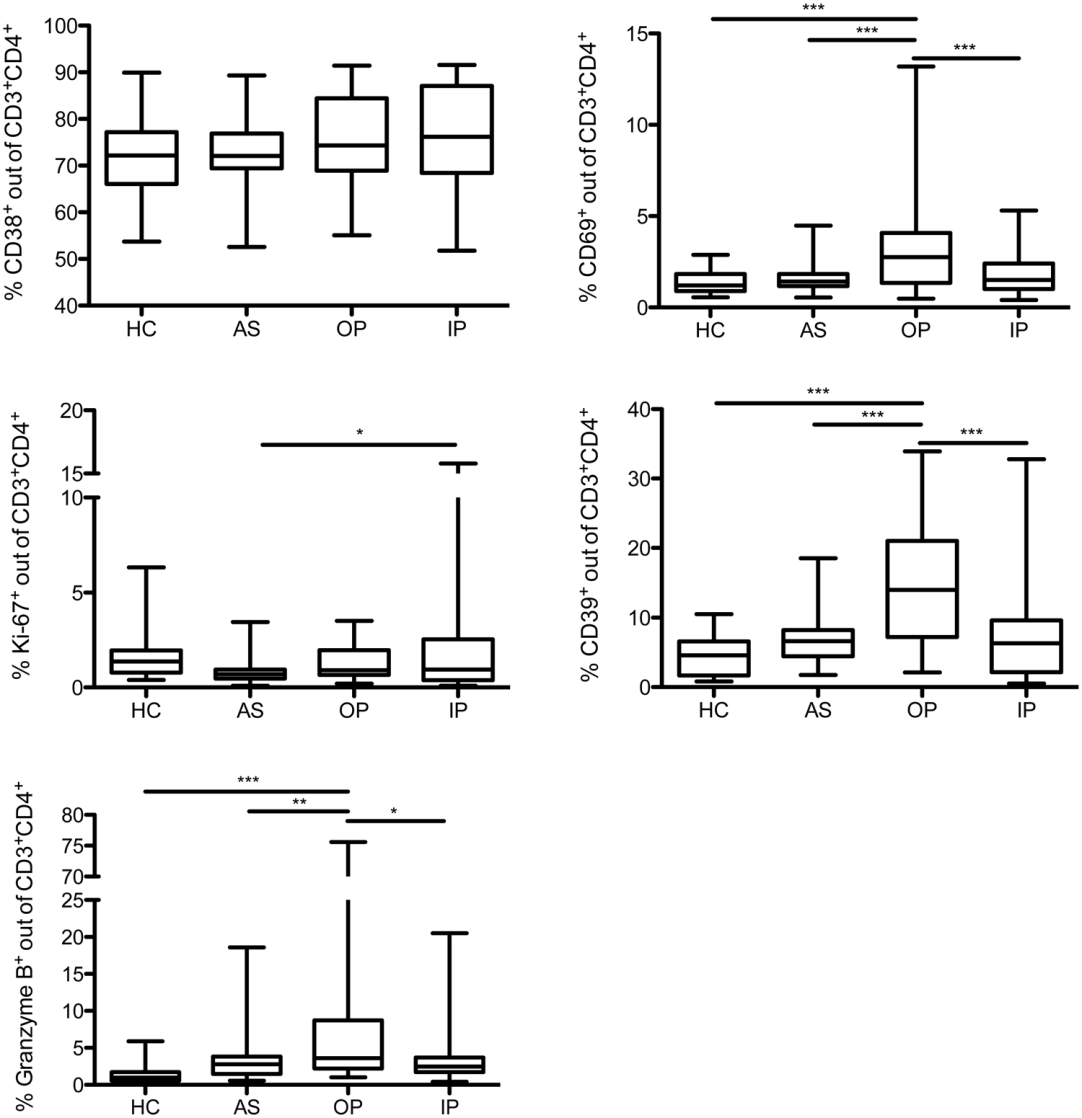
Uncomplicated malaria is associated with a higher expression of CD69, CD39, and Granzyme B on CD4^+^ T cells. Blood samples from patients and controls were *ex vivo* analyzed for the expression of CD38, CD69, CD39, and intracellular Ki-67 and Granzyme B on CD4^+^ T cells by flow cytometry. Box plots demonstrating expression of CD38, CD69, Ki-67, CD39, and Granzyme B on CD4^+^ T cells. Potential statistically significant differences were determined by One-way ANOVA with Tukey’s Multiple Comparison Test. (CD38, CD39, CD69, and GrzB: HC n = 39; AS n = 40; OP n = 27; IP n = 31; Ki67: HC n = 37; AS n = 40; OP n = 19; IP n = 31).

We further measured the expression of other molecules associated with regulatory function, such as the ectoenzymes CD39 and CD38 as well as Granzyme B (GrzB) and the markers for recent activation and proliferation, CD69 and Ki-67, on CD4^+^ T cells in our four study groups. Contrary to the observations with co-inhibitory molecules, the expression of these markers was not overall increased in the two children groups with acute malaria. Only children with uncomplicated malaria, but not children with complicated malaria, showed an increase in the proportion of CD39^+^, CD69^+^, and GrzB^+^ CD4^+^ T cells (Fig. 3). The frequency of Ki-67^+^CD4^+^ T cells was only selectively increased in inpatient treated children, compared to asymptomatically infected children (Fig. 3). For CD38, no difference in expression could be detected between all four groups (Fig. 3).

### No differences in plasma cytokine levels between children with complicated and uncomplicated malaria

**Figure 4:**
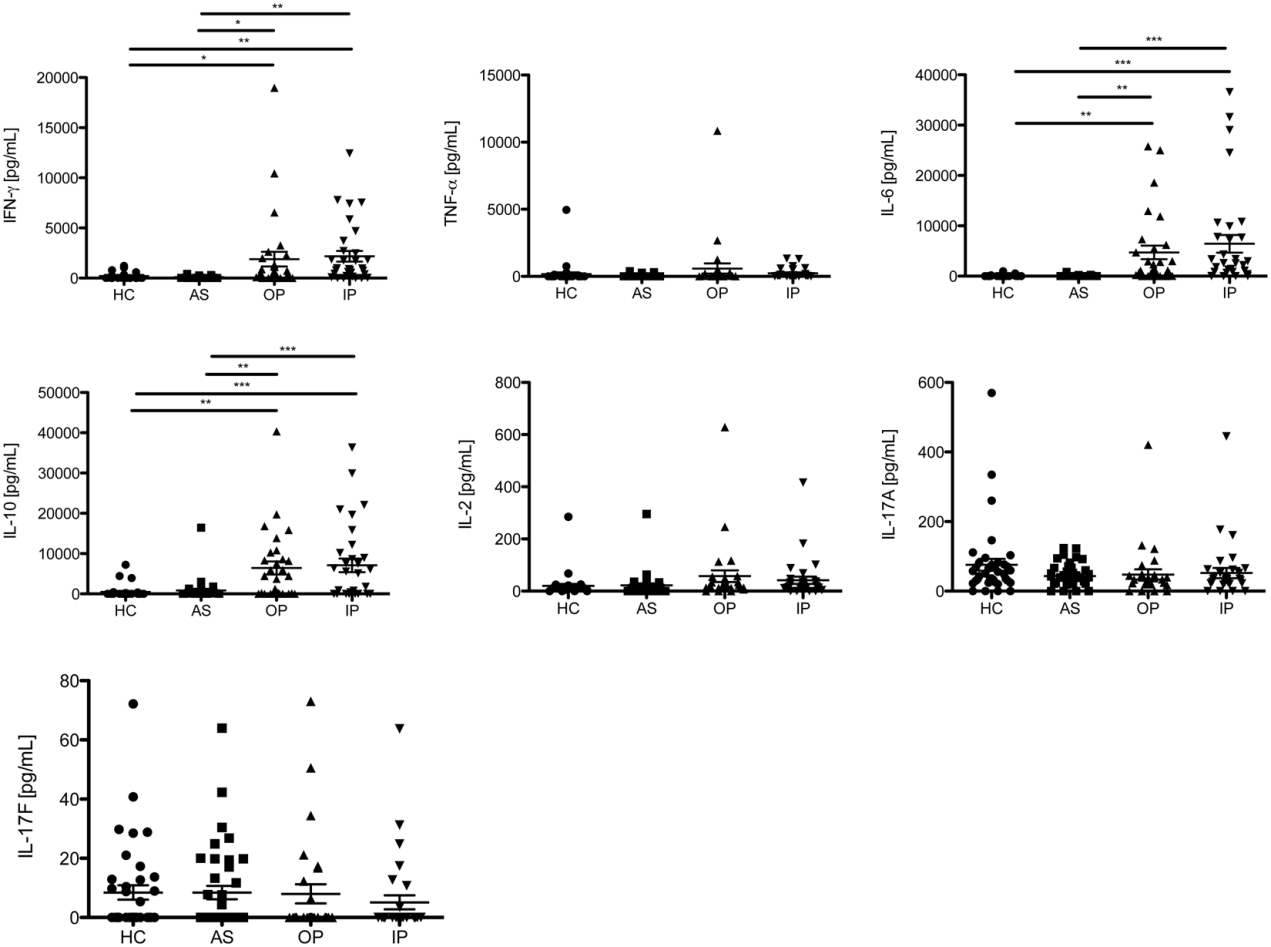
Levels of plasma cytokines do not differ between the outpatient and inpatient group. Plasma samples from patients and controls were *ex vivo* analyzed for the presence of cytokines by LEGENDplex. Scatter plots showing levels of Interferon (IFN)-γ, Interleukin-6 (IL-6), IL-10, IL-2, IL-17F, IL-17A and Tumor necrosis factor (TNF)-α in the four study groups. One-way ANOVA with Tukey’s Multiple Comparison Test was conducted to determine potential statistical differences. (HC n = 38; AS n = 39; OP n = 29; IP n = 32).

Plasma levels of the cytokines IFN-γ, IL-10, TNF-α, IL-6, IL-2, IL-17A, and IL-17F were compared between the four study groups (Fig. 4). Levels of IFN-γ, IL-10, and IL-6 were higher in children with acute malaria, compared to the school children but no difference between children with complicated and uncomplicated malaria could be detected. TNF-α, IL-2, IL-17A, and IL-17F could only be measured in a small number of patients (Fig. 4). A correlation matrix revealed a correlation between IL-2 and IL-17F with TNF-α in the OP group, but no other cytokine correlations were detected in the malaria patients and *Pf* infected children (Supplemental Table 2).

## Discussion

Our study aimed to identify CD4^+^ T cell signatures that were associated with protection from severe and/or clinical malaria. To this end, we assessed the expression pattern of co-inhibitory molecules and other molecules implicated in regulatory capacity on CD4^+^ T cells between children with complicated malaria, uncomplicated malaria, asymptomatic *Pf* infections, and a non-infected control group.

First, we observed a strong increase of CD4^+^ T cells expressing the co-inhibitory molecules CTLA-4, PD-1, LAG-3, and Tim-3 in children with acute malaria, compared to afebrile, healthy children. The highest frequencies of CTLA-4^+^ and PD-1^+^CD4^+^ T cells were found in children with complicated malaria, supporting prior observation with children in endemic areas and adult malaria patients^18,26^.

An addition to standard flow cytometry analysis, we used an automated multivariate analysis to assess the expression pattern of co-inhibitory molecules. An automated approach to flow cytometry data offers the advantage of high reproducibility and excludes subjectivity or bias. On the other hand, rare populations might be lost compared to univariate analyses. Here, results of our univariate approaches could be confirmed by the multivariate analysis which identified a cluster group of T cells which was more frequent in children with complicated malaria than uncomplicated malaria and showed increased expression of CTLA-4, PD-1 and partially LAG-3.

CTLA-4 and PD-1 are usually expressed after T cell receptor-antigen interaction^27,28^ and expression levels here correlated moderately with parasitemia. The higher frequencies of PD-1^+^ and CTLA-4^+^ CD4^+^ T cells are therefore most likely a consequence of the very strong T cell activation in complicated malaria, which then contributes to the more severe symptoms of the patients^29^. This is consistent with observations in other acute infectious diseases such as Ebola, where high levels of CTLA-4 and PD-1 have been associated with high viral loads and fatal outcomes^30^. These observations suggest that a strong T cell receptor activation leads to a compensatory downregulation of the CD4^+^ T cell effector functions through regulatory mechanisms including expression of CTLA-4 and PD-1^17^. Whereas this might help to reduce pathologic effects of a strong adaptive immune response, it probably also contributes to a failure to control the infectious pathogen, here parasitemia, and possibly impairs the induction of malaria-specific memory.

PD-1, CTLA-4 and LAG-3 have also repeatedly been associated with regulatory T cell subsets in different disease models^19,22,31^. Recently, we identified a population of CD4^+^ T cells in acute malaria which showed potent regulatory activity *in vitro* and expressed high levels of CTLA-4 and PD-1^18^. The here identified T cell cluster which was more abundant in children with complicated malaria showed an increased expression of CTLA-4, PD-1 and partially LAG-3, but did not belong to the group with the highest expression levels of these co-inhibitory molecules. Interestingly, T cell clusters with the highest expression of co-inhibitory molecules were not different in patients suffering from uncomplicated or complicated malaria. It is tempting to speculate that CD4^+^ T cell with a high expression of CTLA-4 and PD-1 represent “exhausted” T cells, whereas an intermediate expression might be associated with an immune regulatory phenotype.

Interestingly, we also found a lower expression of CD69, a marker of recent activation, on T cells, in the children group with complicated malaria. So despite a strong prior activation of T cells, which resulted in high levels of CTLA-4 and PD-1, more recent activation and CD69 expression might be down-regulated by expression of these co-inhibitory molecules.

In contrast to the findings with an increased expression of CTLA-4 and PD-1, the frequencies of GrzB^+^ or CD39^+^ CD4^+^ T cells were selectively increased in children with uncomplicated malaria, compared to children with complicated malaria.

An increase of GrzB^+^ CD4^+^ T cells has been repeatedly observed in malaria. Cytotoxic CD4^+^ T cells are induced through immunization and associate with reduced parasitemia or protection after sporozoite rechallenge^32–34^. The mode of protection remains unclear and a potential immunoregulatory role of GrzB^+^ CD4^+^ T cell subsets has been discussed in malaria and other infections^34,35^.

The ectoenzyme CD39 is of particular interest in malaria due to the increased ATP production during infection. CD39 counterbalances ATP-induced pro-inflammatory responses by hydrolyzing ATP to adenosine, which displays an inhibitory effect on T effector cells^36^. ATP is known to be strongly released by infected RBCs during malaria disease and has also been suggested to facilitate parasite invasion and red blood cell adhesion^37,38^.

The increased expression of CD39 and GrzB in children with uncomplicated malaria suggests a protective effect of CD4^+^ T cells expressing these markers regarding the development of malaria-associated complications. This protective effect could result from an improved control of parasitemia and/or from the downregulation of a strong inflammatory response which controls the clinical symptoms but might also hamper immunity.

We initially anticipated that children with asymptomatic *Pf* infection would show an increased expression of regulatory molecules, resulting in a complete control of clinical symptoms. Yet to our surprise, asymptomatically infected children did not show any significant upregulation of co-inhibitory molecules nor GrzB, CD39 or CD69, compared to non-infected children. This also applied to the subgroup of microscopically (thin smear) positive, asymptomatic children. Multiple factors are involved in the induction of these markers during an acute infection. However, it is known that the expression of CTLA-4 is generally induced upon T cell receptor activation^27^ and CTLA-4 levels remained low in the children with asymptomatic *Pf* infection. We therefore propose that the T cell activation levels in these children remain beneath a certain threshold that is required for the induction of co-inhibitory molecule expression. This diminished T cell activation is associated with the asymptomatic clinical picture of the *Pf* infection in this group. This supports the notion that a strong T cell activation during malaria is an important trigger of the clinical symptoms of malaria.

Due to the descriptive, cross-sectional nature of our study, factors influencing the expression of the investigated co-inhibitory molecules could not be fully elucidated. Age groups did not differ in the expression pattern of co-inhibitory molecules. CTLA-4 and PD-1 but none of the other markers correlated moderately with parasitemia. The type of anti-malarial treatment had no effect as the T cell analysis was conducted before initiation of treatment. The duration of the ongoing infection might be an influencing factor that we cannot exclude, which is a limitation of the study. We know that levels of CTLA-4, PD-1, Tim-3 or GrzB expression further increase after initiation of treatment^18^ (and unpublished observation) and a longer time elapse between onset of disease and hospital presentation could be a cause for higher CTLA-4 and PD-1 expression and severer symptoms in the inpatient group. However, the higher frequency of GrzB^+^ CD4^+^ T cells in children with mild malaria argues against purely kinetic reasons for the differential expression pattern of inhibitory molecules in both groups. High levels of pro-inflammatory cytokines have also been proposed to induce CTLA-4^+^ and PD-1^+^ CD4^+^ T cells^30,39^. But we did not detect any differences in plasma cytokine levels in our two acute malaria groups nor did cytokine levels correlate with expression of any of the co-inhibitory molecules.

The similar cytokine levels in our two groups with acute malaria are in contrast to several prior studies that observed differences in pro-inflammatory and anti-inflammatory cytokine levels and their ratio between patients with mild and severe malaria^40–42^. Specifically, we would have expected higher levels of IL-10 in our group with uncomplicated malaria. But measurement of cytokines in plasma is a very broad and unspecific method, influenced by different subsets of leukocytes and their cytokine production. A more specific approach is the intracellular staining of cytokines. In a prior study with adult malaria patients, we detected IFN-γ^+^/IL-10^+^ and IL-10-single positive CD4^+^ T cells which expressed co-inhibitory molecules in a T cell subset that exerted suppressor function *in vitro*. Yet, percentages of T cells with *Pf*-specific cytokine production were very low^18^. It would be of interest to further clarify the function and *Pf*-specific cytokine production of the different CD4^+^ T cell populations identified in this study.

In conclusion, our study results support the hypothesis that the quality of the CD4^+^ T cell response is an important factor influencing the clinical outcome of malaria. Our results identify specific clusters of CTLA-4^+^PD-1^+^CD4^+^ T cells which are associated with malaria complications. Additional longitudinal and functional studies are urgently needed to further investigate if and how CTLA-4^+^PD-1^+^CD4^+^ T cells display a detrimental effect in malaria or rather prevent a worse course of disease.

## Material and Methods

### Study population

The study was conducted as a cross-sectional study in the village Jachie-Pramso in the Bosomtwi District, Ashanti Region, Ghana, an area with perennial malaria transmission, between June-August 2015. In total, 82 healthy children and 68 children with acute malaria between 1–12 years of age were enrolled at two different study sites, Jachie D/A Primary school and St. Michael’s Catholic Hospital, Pramso. At the primary school, 82 healthy, afebrile children were recruited and divided into two subgroups. The first subgroup included 41 children who tested negative in a HRP2 rapid diagnostic test for *Pf* malaria (healthy controls = HC). The remaining 41 children in the second subgroup tested positive in the rapid diagnostic test but were healthy and afebrile and therefore labeled as asymptomatically infected children (AS). Recent malaria treatment or hospital visits were excluded via questionnaire. Children with acute malaria were enrolled at St Michael´s Hospital at time of diagnosis and before initiation of treatment. Malaria patients were also divided into two subgroups. One subgroup included children with fever and mild symptoms who were treated as outpatients with oral artemisinin combination drugs for uncomplicated malaria (“outpatients” = OP). The second group comprised of children with more severe symptoms, that required inpatient treatment with iv Artesunate and were clinically diagnosed with complicated malaria according to the WHO bedside clinical classification of severe malaria (“inpatients” = IP)^43^.

Blood was drawn into heparinized tubes (1–2 mL) before initiation of treatment or directly at the school and processed within 5 hours of collection for *ex vivo* flow cytometry staining.

### Determination of *Plasmodium falciparum* infection

Present *Pf* infections were determined using CareStart™ Malaria HRP2 (PF) rapid diagnostic test. Implementation was carried out according to the manufacturer’s protocol.

Parasitemia was determined with thin blood smears stained with 4% Giemsa and examined under oil immersion (original magnification x100).

### Cell phenotyping by flow cytometric assay

100 μL of whole blood was incubated for 30 minutes at 4 °C with the following surface antibodies: PD-1 PerCP/Cy5.5 (clone EH12.2H7), CD8 AF700 (RPA-T8), Tim-3 BV421 (F38–2E2), CD4 BV510 (OKT4), CD69 FITC (FN50), CD38 PE (HB-7), CD39 BV421 (A1) (all BioLegend), and LAG-3 APC (3DS223H) (eBioscience). Subsequently, the samples were lysed and fixed for 15 minutes at room temperature using RBC Lysis/Fixation Solution (BioLegend) and washed with cold PBS. Thereafter, samples were stained intracellularly with Ki-67 PE/Cy7 (clone Ki-67), CTLA-4 PE (L3D10), CD3 APC/Cy7 (HIT3a), and Granzyme B AF647 (GB11) (all BioLegend) using Foxp3/Transcription Factor Staining Buffer Set (eBioscience) according to the manufacturer’s protocol. After washing, samples were stored at 4 °C until reading on a LSR II Cytometer (4 laser, Becton Dickinson). Where possible, 100 000 cells were collected in the lymphocyte gate. Results were analyzed using FlowJo X 10.0.7r2 (Treestar). Cells were first gated on CD3, followed by a forward/side scatter to exclude cell debris. CD3^+^ T cells were further gated for single cells and the expression of CD4 and CD8. Finally, gates were set on the co-inhibitory molecules and other molecules implicated in regulatory function mentioned above. Fluorescence minus one (FMO) controls were used for setting the gates. The gating strategy and exemplary flow cytometry stainings can be seen in Supplementary Figures S4 and S5. Of note, due to small and variable amounts of blood samples, not all stainings could be conducted in all study participants, which results in different n-values for the expression of the different T cell molecules.

### Multivariate analysis of flow-cytometry data using CITRUS

The expression of the co-inhibitory molecules PD-1, CTLA-4, LAG-3, and Tim-3 was additionally analyzed by the multivariate approach CITRUS implemented in Cytobank^44^. CITRUS enables an automated discovery of statistically significant, differing cell signatures between two groups. By unsupervised hierarchical clustering, cell clusters are identified, phenotypes of the cells are characterized, and differences between groups are determined. Flow cytometry data of children with complicated and uncomplicated malaria were analyzed after using the same gating strategy as before (Supplemental Figure S5). As clustering channels, PD-1, CTLA-4, LAG-3 and Tim-3 were chosen. The association model PAMR with cv_1se was used in abundance mode, and a minimum cluster size of 1 was set in order to consider rare populations. A 10-fold Cross validation was performed and a false discovery rate of 0.01 was applied.

### Plasma cytokine levels

Levels of cytokines in plasma were determined using LEGENDplex™ Human Th Cytokine Panel (13-plex) (BioLegend) according to the manufacturer’s protocol. Samples were read on a BD Accuri™ C6 Flow Cytometer.

### Statistics

The statistical significance of differences between the four groups was defined using One-way ANOVA with Tukey’s Multiple Comparison Test (Graph Pad Prism 5). Correlation analysis was conducted using Pearson Correlation Test. The correlation matrices were composed using Excel correlation analysis. * was used when P <0.05, ** for P <0.01 and *** for P <0.001 unless stated otherwise.

### Study approval

Ethical approval was obtained from the Committee on Human Research, Publication and Ethics, School of Medical Sciences/Komfo Anokye Teaching Hospital, Kwame Nkrumah University of Sciences and Technology, Kumasi, Ghana. Written informed consent was given from a legal guardian/parent of all participants prior to inclusion in the study. The study was carried out in accordance with the principles laid down in the Declaration of Helsinki.

### Data availability

All data generated or analysed during this study are included in this published article (and its Supplementary Information files).

## Electronic supplementary material

Supplemental information
