# Author Correction: Differential expression pattern of co-inhibitory molecules on CD4^+^ T cells in uncomplicated versus complicated malaria

**DOI:** 10.1038/s41598-018-36410-3

**Published:** 2018-11-29

**Authors:** Annemieke Abel, Christiane Steeg, Francis Aminkiah, Otchere Addai-Mensah, Marylyn Addo, Nicola Gagliani, Christian Casar, Denis Dekugmen Yar, Ellis Owusu-Dabo, Thomas Jacobs, Maria Sophia Mackroth

**Affiliations:** 1 Protozoa Immunology, Bernhard Nocht Institute for Tropical Medicine, 20359 Hamburg, Germany; 2 Kumasi Centre for Collaborative Research in Tropical Medicine, Kumasi, Ghana; 3 Department of Medical Laboratory Technology, Faculty of Allied Health Sciences, Kwame Nkrumah University of Science and Technology, Kumasi, Ghana; 4 I. Medical Department, Division of Tropical Medicine and Infectious Diseases, University Medical Centre Hamburg Eppendorf, 20246 Hamburg, Germany; 5 German Center for Infection Research (DZIF), partner site Hamburg-Lübeck-Borstel-Riems, Hamburg, Germany

Correction to: *Scientific Reports* 10.1038/s41598-018-22659-1, published online 19 March 2018

The Acknowledgements section in this Article is incomplete.

“We thank Eric Fomevor, Isaac Yaw Asamoah and Evans Gawu for their technical and logistical help during the study. We especially thank the children and their parents at St Michael’s Hospital and Jachie Primary School for participating in the study.”

should read:

“We thank Eric Fomevor, Isaac Yaw Asamoah and Evans Gawu for their technical and logistical help during the study. We especially thank the children and their parents at St Michael’s Hospital and Jachie Primary School for participating in the study. The work was funded by grants of the Collaborative Research Center 841 of the German Research Foundation and the Werner-Otto Foundation, Hamburg, Germany.”

