# Supplementary material for: Differential expression pattern of co-inhibitory molecules on CD4+ T cells in uncomplicated versus complicated malaria
Source: Sci Rep. 2018 Mar 19;8:4789. doi: 10.1038/s41598-018-22659-1 (PMC5859076; doi:10.1038/s41598-018-22659-1)
Supplement: Supplementary file 1 — Supplemental information [file 41598_2018_22659_MOESM1_ESM.pdf]

Supplemental information

Differential expression pattern of co-inhibitory molecules on $CD4^+$ T cells in uncomplicated versus complicated malaria

Annemieke Abel, Christiane Steeg, Francis Aminkiah, Otchere Addai-Mensah, Marylyn Addo, Nicola Gagliani, Christian Casar, Denis Dekugmen Yar, Ellis Owusu-Dabo, Thomas Jacobs, Maria Sophia Mackroth

Suppl. Tab. 1: Detailed information about study participants, separated according to the four study groups

A

ID	Group	age	sex	rapid test	microscope	parasitemia
1	OP	6	male	positive	positive	0.6
2	OP	9	female	positive	positive	2.7
3	OP	3	male	positive	positive	0.5
4	OP	1	female	positive	positive	0.3
5	OP	9	female	positive	positive	0.6
6	OP	6	male	positive	-	-
7	OP	8	male	positive	positive	0.6
8	OP	4	male	positive	positive	0.1
9	OP	6	female	positive	-	-
11	OP	4	male	positive	Positive	5.7
12	OP	6	female	positive	-	-
13	OP	2	male	positive	positive	0.7
14	OP	6	female	positive	positive	0.3
15	OP	5	female	positive	positive	2.6
16	OP	8	female	positive	positive	1.5
17	OP	4	female	positive	positive	0.7
18	OP	2	male	positive	positive	1.5
19	OP	9	male	positive	positive	2.7
20	OP	8	female	positive	positive	4.5
21	OP	7	female	positive	positive	1.3
22	OP	2	male	positive	-	-
23	OP	3	female	positive	-	-
24	OP	5	female	positive	-	-
25	OP	2	female	positive	-	-
26	OP	9	female	positive	positive	2.5
27	OP	5	male	positive	positive	0.2
28	OP	8	female	positive	positive	0.2
29	OP	10	female	positive	positive	1.7
30	OP	2	male	positive	positive	0.8
OPD +1	OP	10	male	positive	-	-
OPD +2	OP	7	female	positive	positive	0.3
OPD +3	OP	5	male	positive	positive	1.8

B

ID	group	age	sex	rapid test	microscope	parasitemia
31	IP	7	female	positive	positive	17.3
32	IP	4	female	positive	positive	9.4
33	IP	1	male	positive	positive	8.8
34	IP	4	male	positive	positive	3.6
35	IP	9	male	positive	positive	5.3
36	IP	9	female	positive	positive	2.5
37	IP	3	female	positive	positive	3.5
38	IP	4	male	positive	positive	2.4
39	IP	2	male	positive	positive	3.6
40	IP	4	male	positive	-	-
41	IP	4	male	positive	positive	0.5
42	IP	6	female	positive	positive	0.2
43	IP	4	female	positive	positive	10.0
44	IP	4	female	positive	positive	2.9
45	IP	7	male	positive	positive	3.8
46	IP	8	female	positive	positive	5.1
47	IP	2	male	positive	positive	4.6
48	IP	2	male	positive	positive	1.6
49	IP	-	-	positive	-	-
50	IP	1	female	positive	positive	1.0
51	IP	6	male	positive	positive	9.9
52	IP	2	female	positive	positive	13.0
53	IP	2	male	positive	positive	2.3
54	IP	10	female	positive	-	-
55	IP	7	male	positive	positive	1.1
56	IP	2	female	positive	-	-
57	IP	3	female	positive	-	-
58	IP	4	female	positive	-	-
59	IP	8	male	positive	positive	0.3
60	IP	1	female	positive	positive	1.8
61	IP	6	male	positive	positive	1.7
62	IP	6	male	positive	-	-
63	IP	5	male	positive	positive	4.9
64	IP	12	female	positive	positive	2.7
65	IP	1	male	positive	positive	1.9

C

ID	group	age	sex	rapid test	microscope	parasitemia
101	HC	10	male	negative	-	-
103	HC	7	male	negative	-	-
104	HC	7	male	negative	-	-
105	HC	6	male	negative	-	-
112	HC	7	female	negative	-	-
113	HC	8	male	negative	-	-
117	HC	8	female	negative	-	-
120	HC	8	female	negative	-	-
122	HC	9	male	negative	-	-
124	HC	10	female	negative	-	-
126	HC	8	female	negative	-	-
129	HC	10	male	negative	-	-
132	HC	9	female	negative	-	-
136	HC	9	female	negative	-	-
137	HC	9	female	negative	-	-
140	HC	8	female	negative	-	-
142	HC	11	female	negative	-	-
150	HC	9	female	negative	-	-
153	HC	9	male	negative	-	-
154	HC	9	male	negative	-	-
158	HC	10	female	negative	-	-
161	HC	9	female	negative	-	-
162	HC	10	female	negative	-	-
164	HC	7	female	negative	-	-
166	HC	6	female	negative	-	-
168	HC	9	male	negative	negative	0
172	HC	8	male	negative	negative	0
173	HC	5	male	negative	-	-
174	HC	6	female	negative	-	-
183	HC	8	female	negative	-	-
193	HC	10	female	negative	-	-
201	HC	9	female	negative	-	-
202	HC	8	female	negative	-	-
205	HC	8	female	negative	-	-
206	HC	9	female	negative	-	-
211	HC	8	female	negative	-	-
219	HC	8	female	negative	-	-
223	HC	10	female	negative	-	-
224	HC	10	male	negative	-	-
227	HC	10	female	negative	-	-
232	HC	9	male	negative	-	-

D

ID	group	age	sex	rapid test	microscope	parasitemia
108	AS	10	male	positive	negative	0.0
109	AS	9	male	positive	positive	0.1
110	AS	10	female	positive	positive	0.4
111	AS	7	female	positive	negative	0.0
114	AS	9	male	positive	positive	0.1
118A	AS	6	male	positive	positive	0.1
118B	AS	9	male	positive	positive	0.2
127	AS	10	female	positive	positive	0.2
128	AS	10	female	positive	positive	0.3
131	AS	10	female	positive	negative	0.0
135	AS	10	female	positive	negative	0.0
138	AS	11	male	positive	negative	0.0
139	AS	11	female	positive	positive	0.3
143	AS	11	female	positive	negative	0.0
144	AS	9	male	positive	positive	0.1
145	AS	9	female	positive	negative	0.0
148	AS	10	female	positive	negative	0.0
152	AS	9	female	positive	negative	0.0
155	AS	11	male	positive	negative	0.0
156	AS	10	male	positive	negative	0.0
159	AS	11	female	positive	negative	0.0
167	AS	9	male	positive	negative	0.0
169	AS	6	female	positive	positive	0.3
170	AS	7	female	positive	positive	0.1
181	AS	8	female	positive	negative	0.0
185	AS	8	female	positive	negative	0.0
189	AS	9	female	positive	negative	0.0
190	AS	10	female	positive	negative	0.0
191	AS	10	female	positive	negative	0.0
195	AS	7	male	positive	negative	0.0
196	AS	8	male	positive	negative	0.0
199	AS	10	female	positive	positive	0.1
200	AS	8	female	positive	positive	0.2
203	AS	8	female	positive	negative	0.0
209	AS	11	male	positive	positive	0.2
212	AS	10	male	positive	negative	0.0
213	AS	10	male	positive	negative	0.0
216	AS	7	female	positive	negative	0.0
218	AS	9	female	positive	negative	0.0
222	AS	10	male	positive	negative	0.0
238	AS	8	male	positive	positive	0.1

Suppl. Tab. 2: Correlarion matrices of plasma cytokines. Data was compiled with EXCEL correlation analysis. Seperated tables for all groups taken together, and all four groups HC, AS, OP, and IP themselves are displayed. Values marked green represent statistically significant correlations between two cytokines confirmed by regression analysis.

**all**	*IFN-γ [pg/mL]*	*TNF-α [pg/mL]*	*IL-6 [pg/mL]*	*IL-10 [pg/mL]*	*IL-2 [pg/mL]*	*IL-17A [pg/mL]*	*IL-17F [pg/mL]*
IFN-γ [pg/mL]	1	0,12	0,17	0,15	0,10	0,15	0,09
TNF-α [pg/mL]	0,12	1	0,03	0,02	0,72	0,21	0,43
IL-6 [pg/mL]	0,17	0,03	1	0,06	0,02	-0,05	-0,01
IL-10 [pg/mL]	0,15	0,02	0,06	1	0,03	0,17	-0,13
IL-2 [pg/mL]	0,10	0,72	0,02	0,03	1	0,10	0,32
IL-17A [pg/mL]	0,15	0,21	-0,05	0,17	0,10	1	0,14
IL-17F [pg/mL]	0,09	0,43	-0,01	-0,13	0,32	0,14	1

**HC**	*IFN-γ [pg/mL]*	*TNF-α [pg/mL]*	*IL-6 [pg/mL]*	*IL-10 [pg/mL]*	*IL-2 [pg/mL]*	*IL-17A [pg/mL]*	*IL-17F [pg/mL]*
IFN-γ [pg/mL]	1	0,50	0,68	0,66	0,44	0,57	0,13
TNF-α [pg/mL]	0,50	1	0,84	0,85	0,96	0,54	0,22
IL-6 [pg/mL]	0,68	0,84	1	0,98	0,73	0,84	0,18
IL-10 [pg/mL]	0,66	0,85	0,98	1	0,75	0,81	0,17
IL-2 [pg/mL]	0,44	0,96	0,73	0,75	1	0,44	0,25
IL-17A [pg/mL]	0,57	0,54	0,84	0,81	0,44	1	0,24
IL-17F [pg/mL]	0,13	0,22	0,18	0,17	0,25	0,24	1

**AS**	*IFN-γ [pg/mL]*	*TNF-α [pg/mL]*	*IL-6 [pg/mL]*	*IL-10 [pg/mL]*	*IL-2 [pg/mL]*	*IL-17A [pg/mL]*	*IL-17F [pg/mL]*
IFN-γ [pg/mL]	1	-0,02	0,33	0,55	-0,11	0,23	0,42
TNF-α [pg/mL]	-0,02	1	-0,11	0,54	0,03	-0,33	0,13
IL-6 [pg/mL]	0,33	-0,11	1	0,04	0,10	0,09	0,26
IL-10 [pg/mL]	0,55	0,54	0,04	1	-0,02	-0,26	0,18
IL-2 [pg/mL]	-0,11	0,03	0,10	-0,02	1	0,06	-0,04
IL-17A [pg/mL]	0,23	-0,33	0,09	-0,26	0,06	1	0,10
IL-17F [pg/mL]	0,42	0,13	0,26	0,18	-0,04	0,10	1

**OP**	*IFN-γ [pg/mL]*	*TNF-α [pg/mL]*	*IL-6 [pg/mL]*	*IL-10 [pg/mL]*	*IL-2 [pg/mL]*	*IL-17A [pg/mL]*	*IL-17F [pg/mL]*
IFN-γ [pg/mL]	1	0,02	-0,09	0,05	0,07	0,14	-0,07
TNF-α [pg/mL]	0,02	1	-0,07	-0,10	0,90	0,09	0,72
IL-6 [pg/mL]	-0,09	-0,07	1	-0,16	-0,15	-0,01	0,10
IL-10 [pg/mL]	0,05	-0,10	-0,16	1	-0,16	0,21	-0,21
IL-2 [pg/mL]	0,07	0,90	-0,15	-0,16	1	0,04	0,75
IL-17A [pg/mL]	0,14	0,09	-0,01	0,21	0,04	1	0,05
IL-17F [pg/mL]	-0,07	0,72	0,10	-0,21	0,75	0,05	1

**IP**	*IFN-γ [pg/mL]*	*TNF-α [pg/mL]*	*IL-6 [pg/mL]*	*IL-10 [pg/mL]*	*IL-2 [pg/mL]*	*IL-17A [pg/mL]*	*IL-17F [pg/mL]*
IFN-γ [pg/mL]	1	0,50	0,07	-0,13	-0,05	0,34	0,55
TNF-α [pg/mL]	0,50	1	0,08	-0,23	-0,02	0,41	0,65
IL-6 [pg/mL]	0,07	0,08	1	-0,17	-0,01	-0,11	-0,03
IL-10 [pg/mL]	-0,13	-0,23	-0,17	1	-0,01	0,29	-0,24
IL-2 [pg/mL]	-0,05	-0,02	-0,01	-0,01	1	0,02	0,04
IL-17A [pg/mL]	0,34	0,41	-0,11	0,29	0,02	1	0,14
IL-17F [pg/mL]	0,55	0,65	-0,03	-0,24	0,04	0,14	1

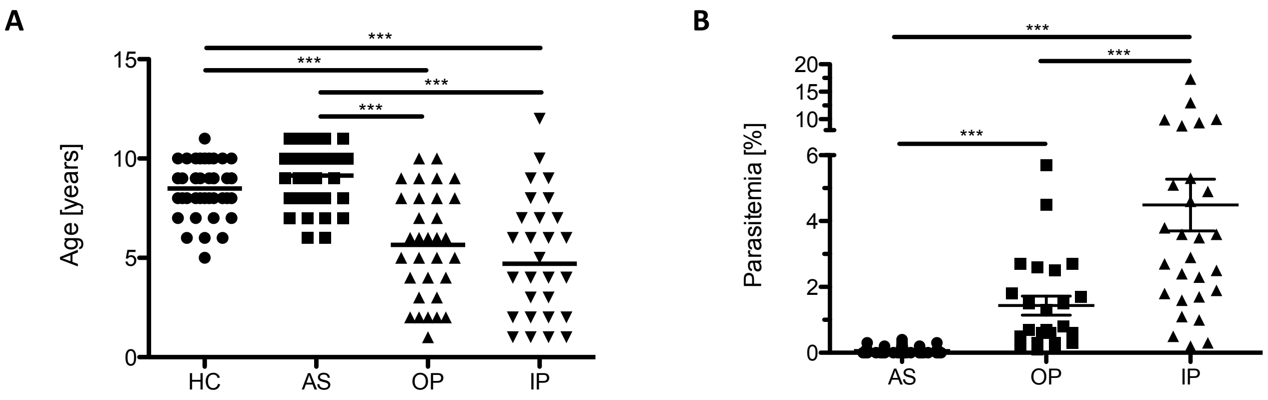

Supplemental Figure S1: Characteristics of study participants. A) Information was given by parents/legal guardians. One-way ANOVA with Tukey's Multiple Comparison Test was conducted to determine potential statistical differences. (HC n= 41; AS n= 41; OP n= 32; IP n= 34) B) Parasitemia was determinded by thin blood smears stained with 4% Giemsa and examined under oil immersion (original magnification x 100). In order to determine statistically significant differences, Mann-Whitney U Test was used. (AS n= 41; OP n= 24; IP n= 28)

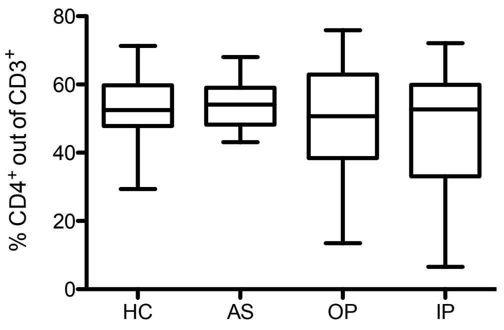

Supplemental Figure S2: No changes in CD4 counts after *Plasmodium falciparum* infection. Blood samples from patients and healthy controls were ex vivo analyzed for the expression of CD3 and CD4 by flow cytometry. Potential statistically significant differences were determined by One-way ANOVA with Tukey's Multiple Comparison Test. (HC n= 37; AS n= 40; OP n= 27; IP n= 31).

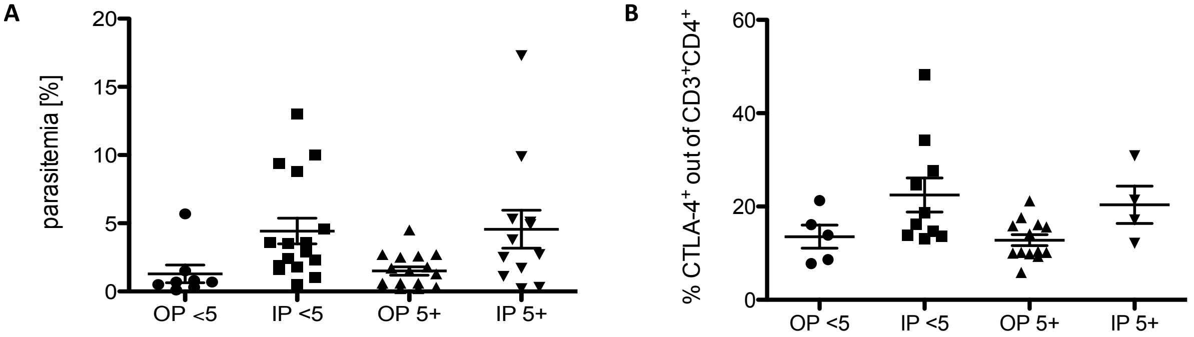

Supplemental Figure S3: No difference in parasitemia nor CTLA-4 expression in $CD4^+$ T cells in children younger than 5 years and children older than 5 years. A) Parasitemia was determinded by thin blood smears stained with 4% Giemsa and examined under oil immersion (original magnification x 100). Analysis by One-way ANOVA with Tukey's Multiple Comparison Test. (OP <5 n= 8; IP <5 n= 16; OP 5+ n= 16; IP 5+ n= 12) B) Whole blood samples from patients and controls were ex vivo analyzed for the expression of CTLA-4 in $CD4^+$ T cells. One-way ANOVA with Tukey's Multiple Comparison Test was conducted to determine potential statistically significant differences. (OP <5 n= 5; IP <5 n= 10; OP 5+ n= 13; IP 5+ n= 4).

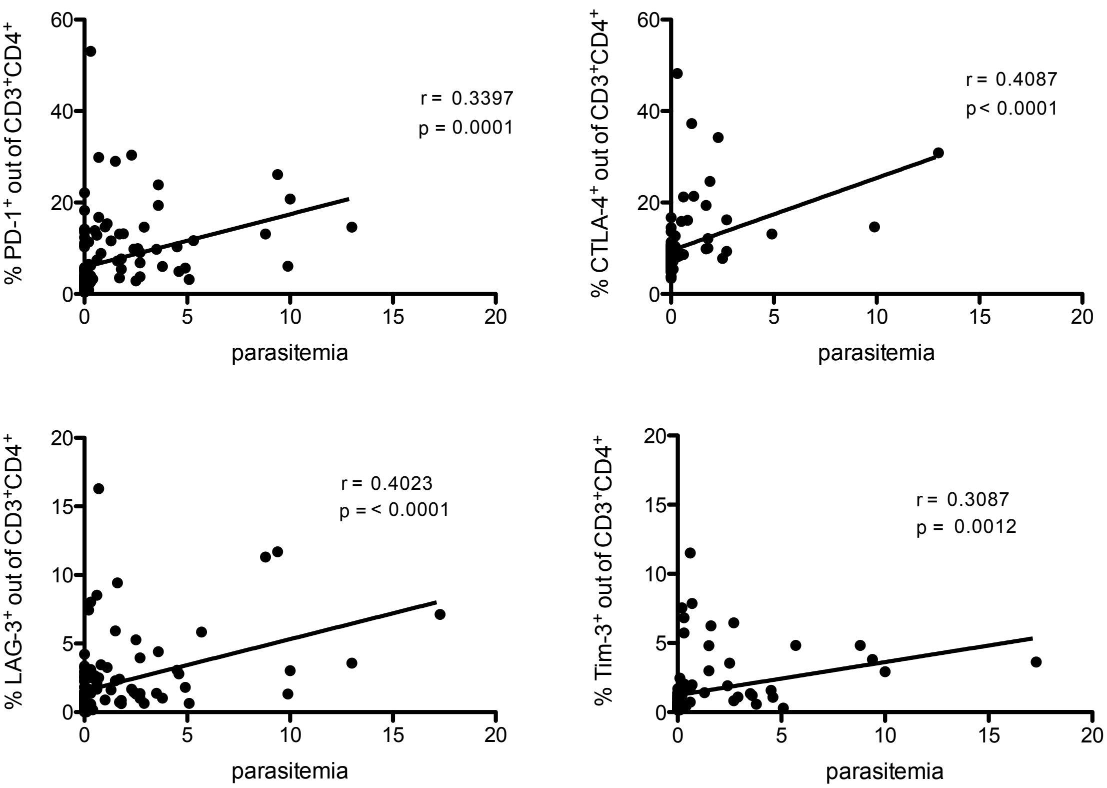

Supplemental Figure S4: Expression of co-inhibitory molecules does not correlate with parasitemia. Percentages of CD4$^+$ T cells expressing one of the co-inhibitory molecules analyzed were plotted against parasitemia of all study groups. Correlation was analyzed with Pearson correlation. (PD-1 n= 120; CTLA-4 n= 80; LAG-3 n= 125; Tim-3 n= 108).

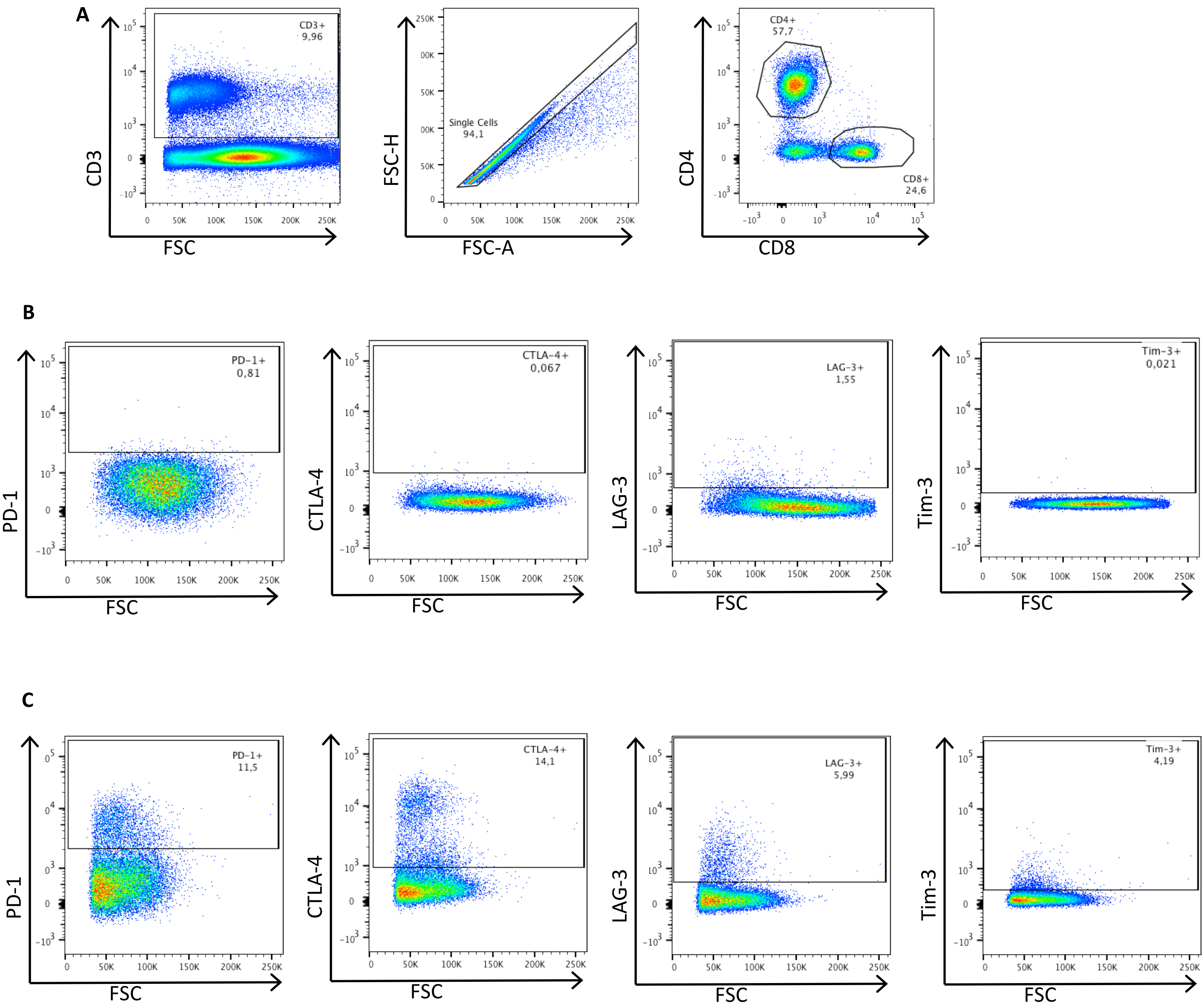

Supplemental Figure S5: Gating strategy and stainings for co-inhibitory molecules. A) Gating strategy gating for $CD3^+$ cells, single cells, and $CD4^+$/$CD8^+$ cells, applied to all flow cytometric analyzes. B) Dotplots for FMO stainings of PD-1, CTLA-4, LAG-3, and Tim-3 on $CD4^+$ T cells. C) Exemplary stainings for the co-inhibitory molecules PD-1, CTLA-4, LAG-3, and Tim-3 for one representative OP child.

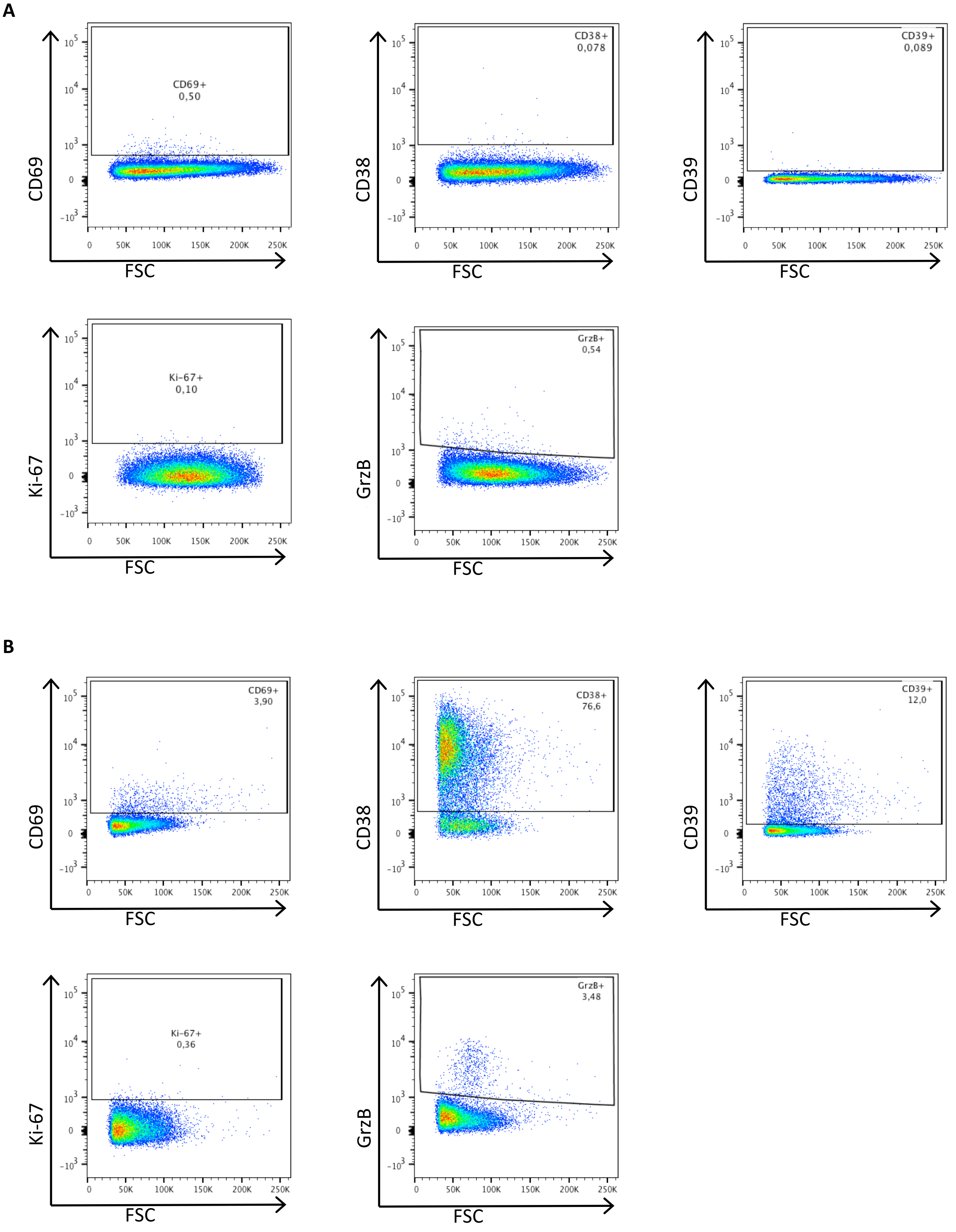

Supplemental Figure S6: Flow cytometry stainings for CD69, CD38, CD39, Ki-67, and Granzyme B on CD4^{+} T cells. A) FMOs of CD69, CD38, CD39, Ki-67, and Granzyme B on CD4^{+} T cells gated as demonstrated in Supplemental Figure S2. B) Dotplots show exemplary staining for CD69, CD38, CD39, Ki-67, and Granzyme B for one representative OP child.
